# Personal

**Published:** 1889-08

**Authors:** 


### Personal.

DR. EDWARD CLARK, City Health Physician, will attend the annual meeting of the American Public Health Association, to be held in Brooklyn, N. Y., October 22, 23, 24, and 25, 1889, as a delegate from the Buffalo Health Department.

THE DEGREE OF DOCTOR OF MEDICINE, *Honoris Causâ*, was conferred upon Dr. William Warren Potter, of this city, by the Kentucky School of Medicine, at its last annual commencement, held in Louisville, June 20, 1889.

DR. W. G. GREGORY, of this city, was elected President of the New York State Pharmaceutical Association at its recent annual meeting held in Binghamton.

